# Supplementary material for: A glucose meter interface for point-of-care gene circuit-based diagnostics
Source: Nat Commun. 2021 Feb 1;12:724. doi: 10.1038/s41467-020-20639-6 (PMC7851131; doi:10.1038/s41467-020-20639-6)
Supplement: Supplementary file 1 — Supplementary Information [file 41467_2020_20639_MOESM1_ESM.pdf]

Supplementary Materials for

A Glucose Meter Interface for Point-of-Care Gene Circuit-based Diagnostics

Authors:
Evan Amalfitano[1]†, Margot Karlikow[1]†, Masoud Norouzi[1]†, Katariina Jaenes[1], Seray Cicek[1], Fahim Masum[1], Peivand Sadat Mousavi[1], Yuxiu Guo[1], Laura Tang[1], Andrew Sydor[2], Duo Ma[3], Joel D. Pearson[4,5,6], Daniel Trcka[4], Mathieu Pinette[7], Aruna Ambagala[7], Shawn Babiuk[7], Bradley Pickering[7,8,9], Jeff Wrana[4,10], Rod Bremner[4,5,6], Tony Mazzulli[6,11], David Sinton[12], John H. Brumell[2,10,13,14], Alexander A. Green[3,15], Keith Pardee[1,12]*

Affiliations:
[1] Leslie Dan Faculty of Pharmacy, University of Toronto, Toronto, ON M5S 3M2, Canada.
[2] Program in Cell Biology, Hospital for Sick Children, Peter Gilgan Center for Research and Learning, 686 Bay Street, Toronto, ON, M5G 0A4, Canada.
[3] Biodesign Center for Molecular Design and Biomimetics, The Biodesign Institute and the School of Molecular Sciences, Arizona State University, AZ 85287, USA.
[4] Lunenfeld Tanenbaum Research Institute, Mt Sinai Hospital, Sinai Health System, Toronto,
Canada.
[5] Department of Ophthalmology and Vision Science, University of Toronto, Toronto, Canada
[6] Department of Laboratory Medicine and Pathobiology, University of Toronto, Toronto, Canada
[7] Canadian Food Inspection Agency, National Centre for Foreign Animal Disease, Winnipeg, Canada
[8] Department of Medical Microbiology and Infectious Diseases, Faculty of Medicine, University of Manitoba, Winnipeg, Canada.
[9] Iowa State University, College of Veterinary Medicine, Department of Veterinary Microbiology and Preventive Medicine, Ames, Iowa, United States of America
[10] Department of Molecular Genetics, University of Toronto, Toronto, Canada
[11] Department of Microbiology, Sinai Health System/University Health Network, Toronto, Canada
[12] Department of Mechanical and Industrial Engineering, University of Toronto, Toronto, ON, M5S 1A1, Canada.
[13] Institute of Medical Science, University of Toronto, Toronto, ON, M5S 1A1, Canada.
[14] SickKids IBD Centre, Hospital for Sick Children, Toronto, ON, M5G 0A4, Canada.
[15] Department of Biomedical Engineering, Boston University, Boston, MA 02215, USA.

* Corresponding author. Email: keith.pardee@utoronto.ca (K.P.)
† Equal contribution.

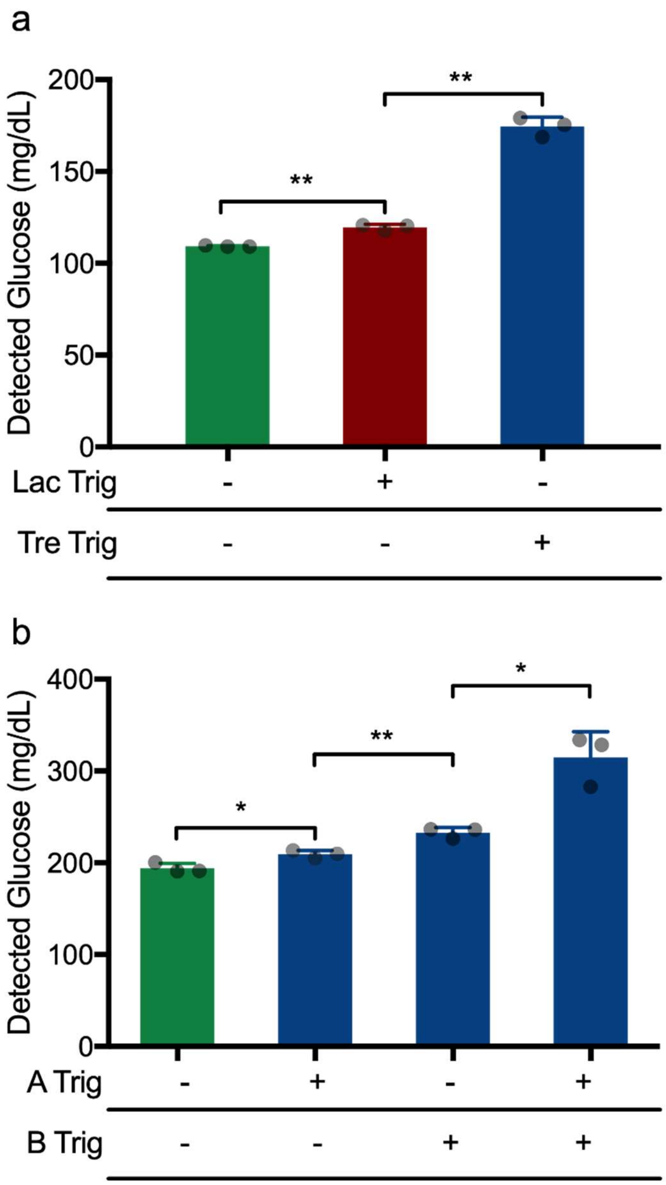

Supp. Fig. 1. Demonstration of glucose signal multiplexing using two different methods. **a** Glucose production from two toehold switches, each with a different reporter enzyme (lactase or trehalase), present in all three reactions. Trigger RNAs for the lactase switch ("Lac") and for the trehalase switch ("Tre") are added at equal concentrations. Lactase switch (Synthetic switch B) present at 17.5 ng/µL, trehalase switch (Synthetic switch A) at 2.5 ng/µL. Trigger RNA at 5 nM. **: Neg vs. Lac Trig p=0.0061, Tre Trig vs. Lac Trig p=0.0014. **b** Similar to **a** except toehold switches (Synthetic switches A and B, 5 ng/µL) express the same trehalase reporter enzyme at different levels. RNA triggers for each switch (A, B or A+B) are added at equal concentration (5 nM). *: Neg vs. A Trig p=0.0208, A + B Trig vs. B Trig p=0.0325; **: p=0.0057. All data presented are the mean of N=3 independent experiments (as indicated by dot plots) +/- SD.

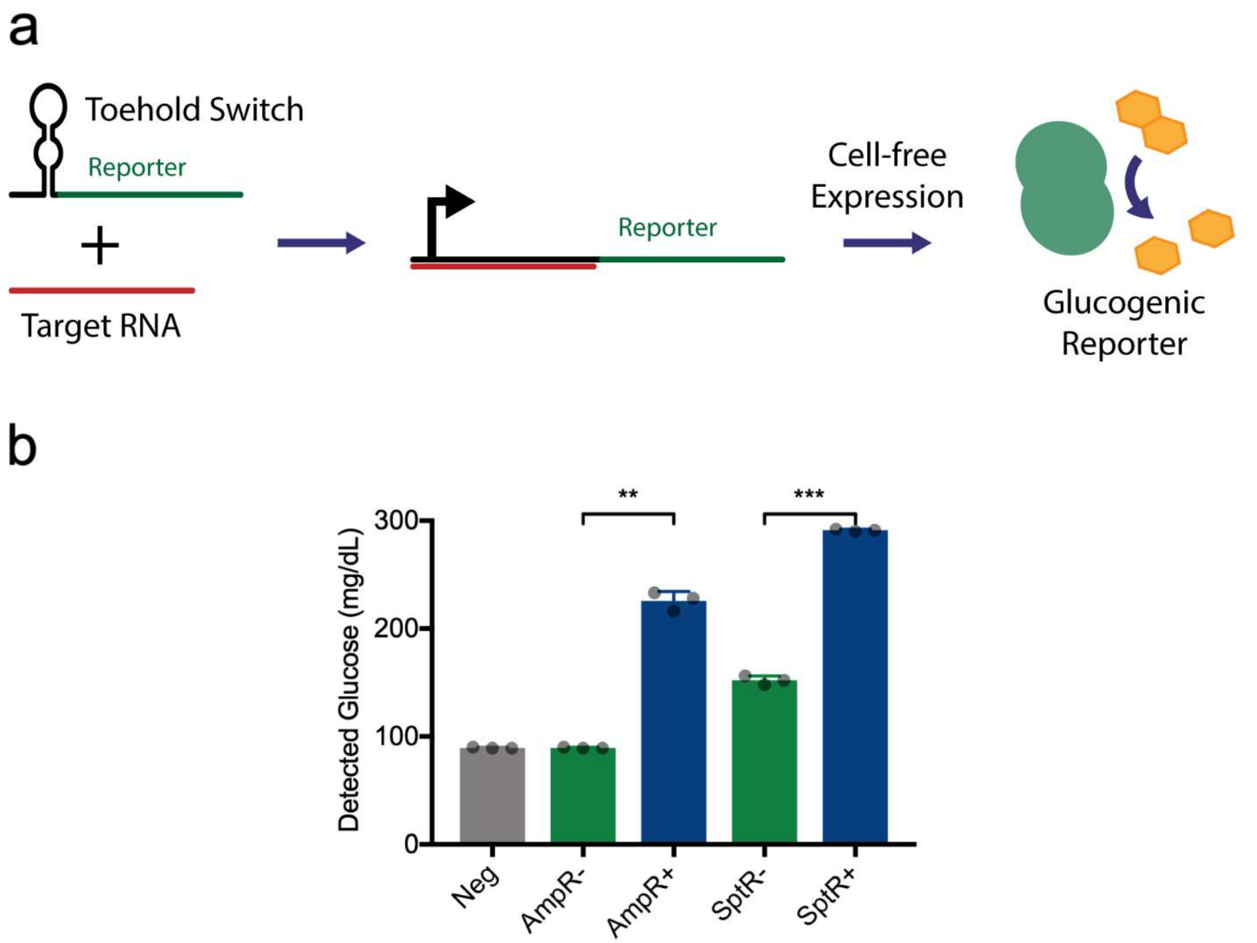

Supp. Fig. 2. Detection of antibiotic resistance genes using toehold switch-based sensors and a glucose meter. **a** A schematic showing the use of a toehold switch-based sensor to regulate gene expression. Toehold switch secondary structure prevents the reporter gene from being translated, but changes conformation in the presence of target RNA with the appropriate sequence, allowing the reporter to be expressed. By using a glucogenic reporter, a glucose meter can be used to read the results. **b** Using appropriate toehold switches and trehalase as the reporter gene, glucose production was detected using a glucose meter. "-" indicates that switch is present but without target RNA, "+" indicates switch and target RNA are both present. AmpR: ampicillin resistance sensor (plasmid DNA template, 1.25 ng/µL), SptR: spectinomycin resistance sensor (linear DNA template, 1.25 ng/µL). **: p=0.0013; ***: p=0.0001. N=3 technical replicates (as indicated by dot plots), representative of 3 independent experiments. Data presented as mean of +/- SD.

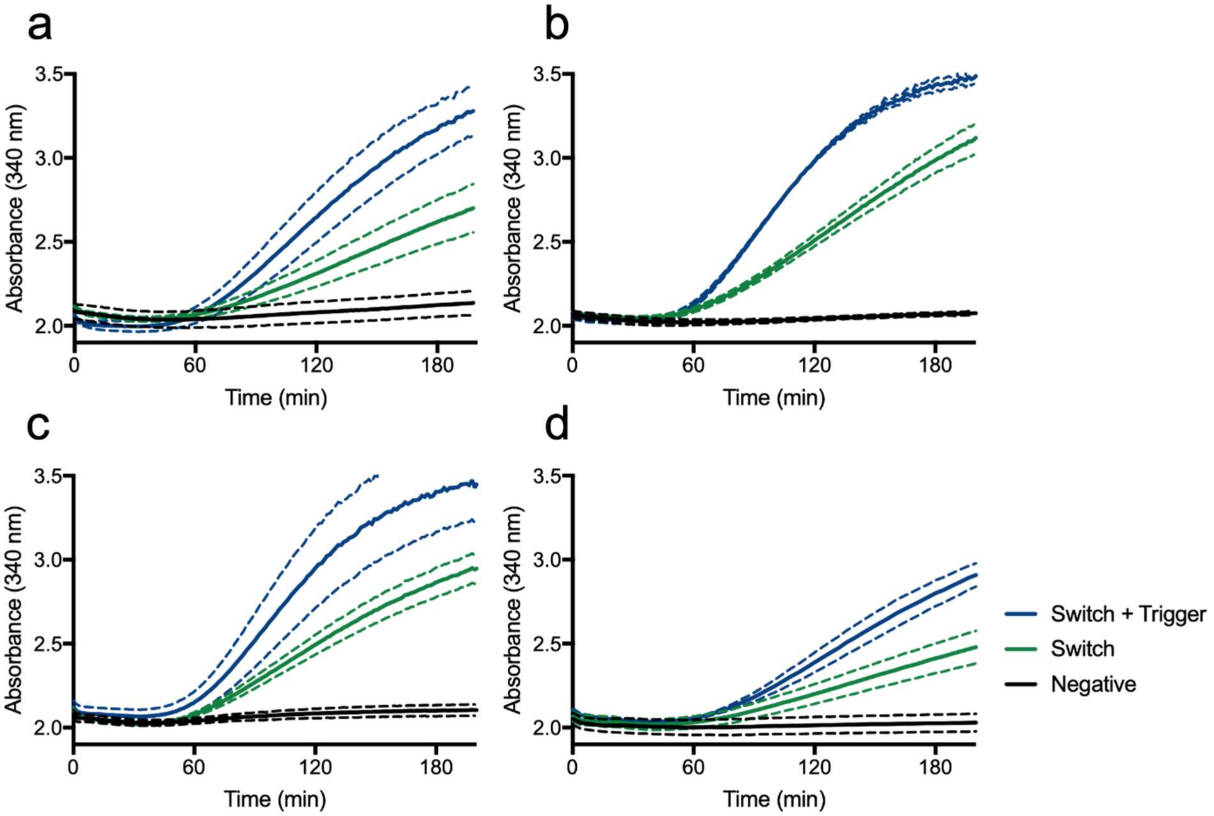

Supp. Fig. 3. Screening results of the four top-performing toehold switches targeting *S. typhi* and related genes using absorbance-based GDH-NAD glucose assay (340 nm). **a** The screening result for the toehold switch chosen as the STY sensor, with the glucose production measured in the presence of the target RNA (Switch + Trigger) and compared to treatments in the absence of target RNA (Switch) and the CFS control without DNA inputs (Negative). **b**, **c**, **d**, The equivalent data for the toehold switches targeting paratyphoid A, paratyphoid B, and fluoroquinolone resistance respectively (labels as in **a**). Data represent the mean absorbance value for N=3 technical replicates, with dotted lines representing SEM.

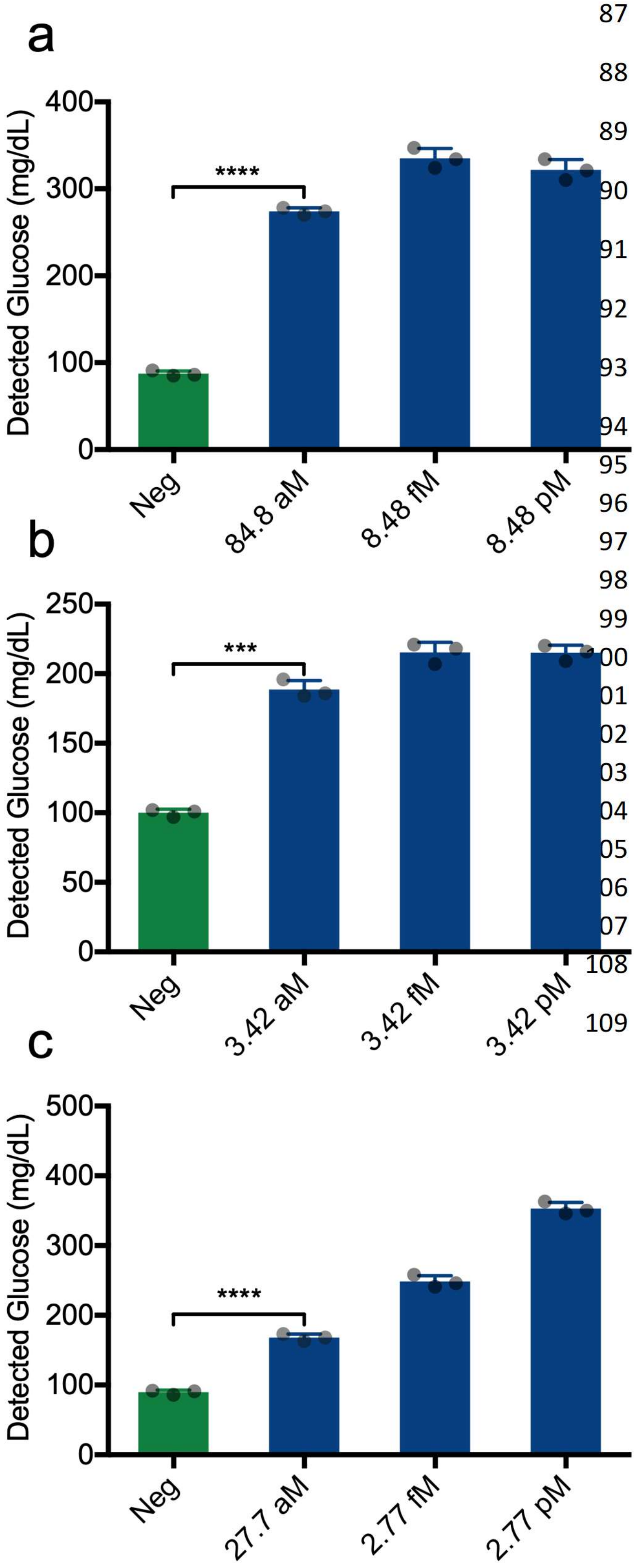

Supp. Fig. 4. Sensitivity tests using NASBA isothermal amplification for toehold switches targeting RNAs from **a** Paratyphoid A, ****: $p<0.0001$, **b** Paratyphoid B, ***: $p=0.0004$, or the **c** fluoroquinolone resistance gene QnrS, ****: $p<0.0001$. Numbers show the concentration of target RNA present in water before being added to the NASBA reaction. N=3 technical replicates (as indicated by dot plots), representative of the results from 3 independent experiments. Data are presented as mean values +/- SD.

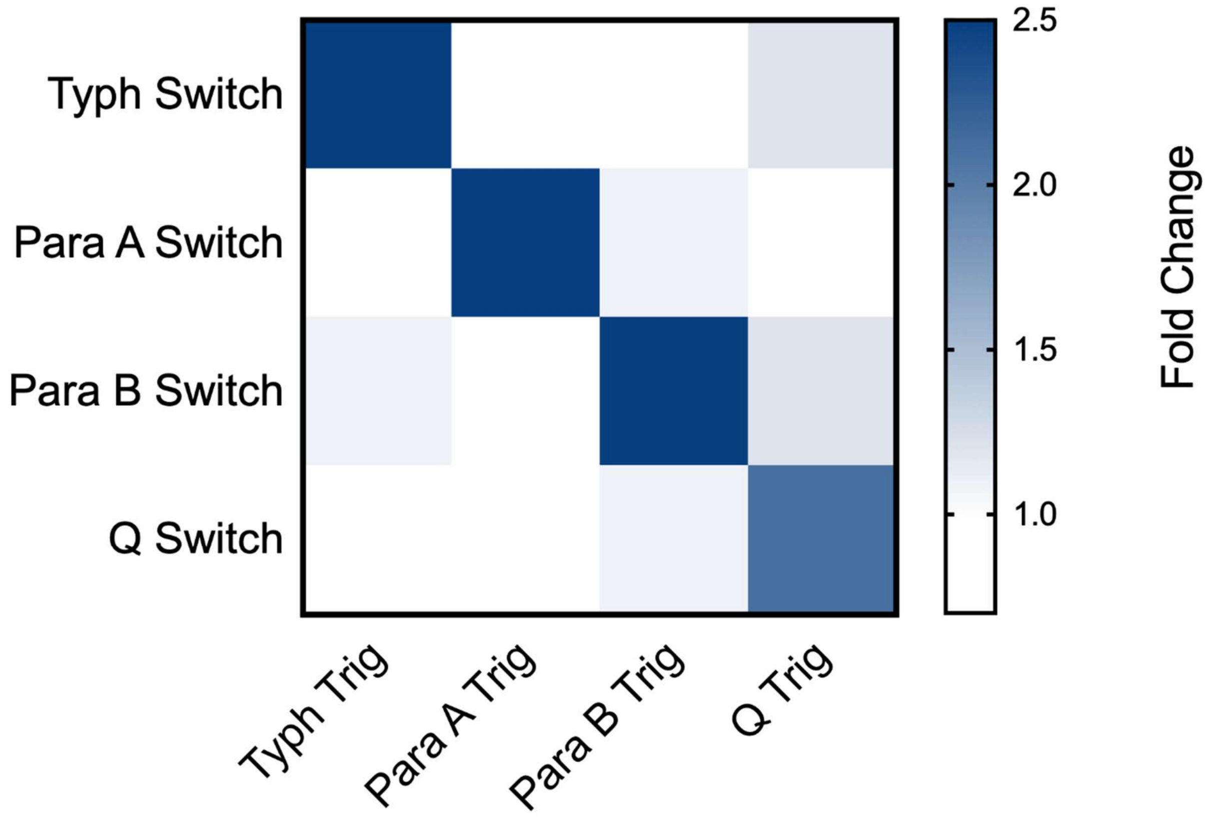

Supp. Fig. 5. Orthogonality screen of typhoid-related toehold switches to evaluate cross-reactivity between sensor and target RNA pairs. The color scale indicates average fold change in glucose generation compared to the average non-specific background signal following 60 minutes of incubation at 37° C. N=3 technical replicates.

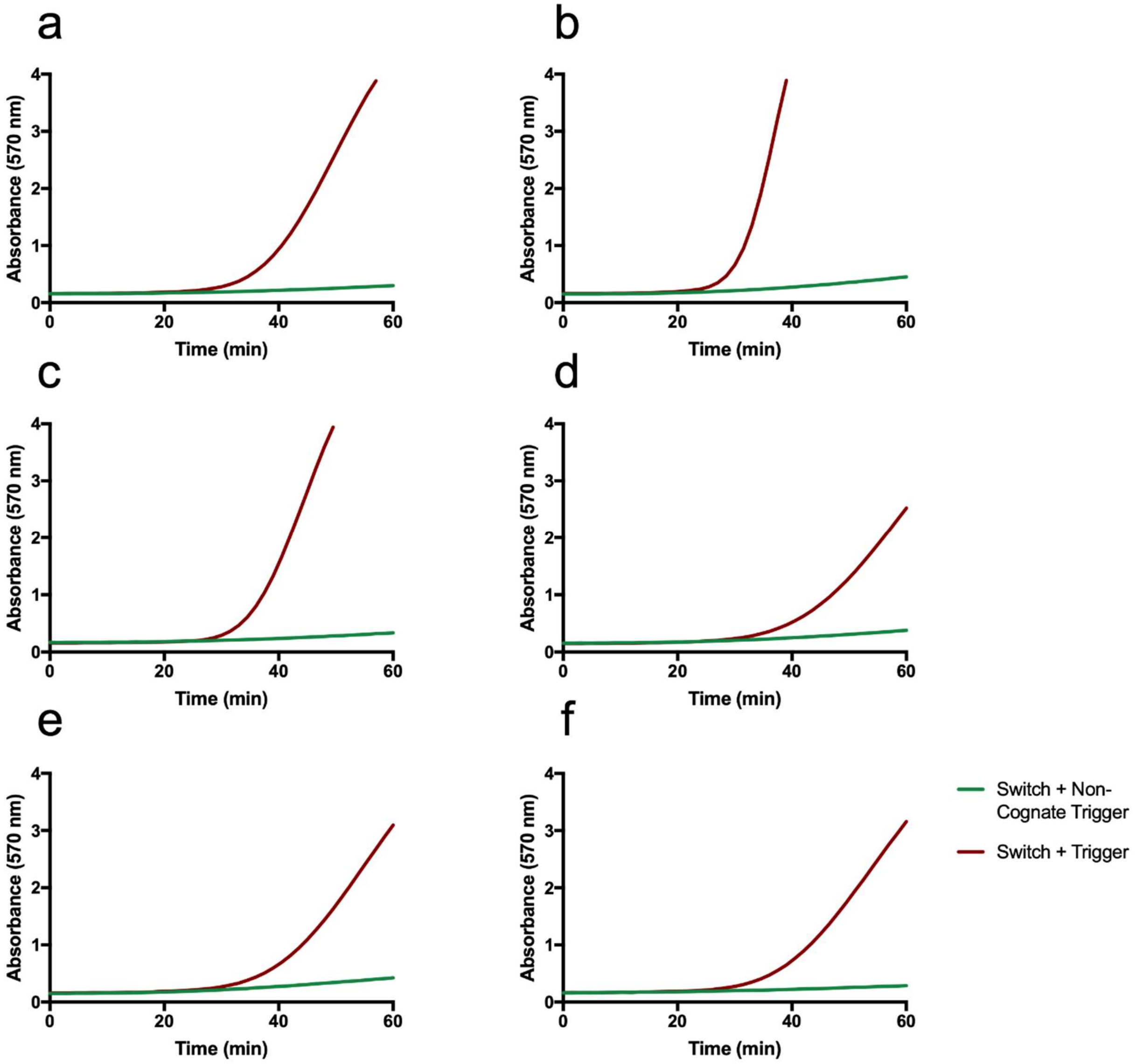

Supp. Fig. 6. Rapid screening of the SARS-CoV-2 toehold switches using an absorbance-based lactase activity assay with CPRG as the colorimetric substrate. The results presented report the optical signal (570 nm) generated in the presence of the corresponding target RNA (brick red line) and in the presence of a non-cognate RNA from MERS (green line). **a** Time course data for CFS reactions containing the RdRP switch D and corresponding trigger, **b**, **c**, **d**, show equivalent data for the corresponding toehold switches **b** E gene switch C (E gene C), **c** E gene switch D (E gene D), **d** Switch B for CDC target region N1 within the N gene, **e** Switch A for CDC target region N3 within the N gene (N3 A), **f** Switch B CDC target region N3 within the gene (N3 B). For all screens, N=1 biological replicate.

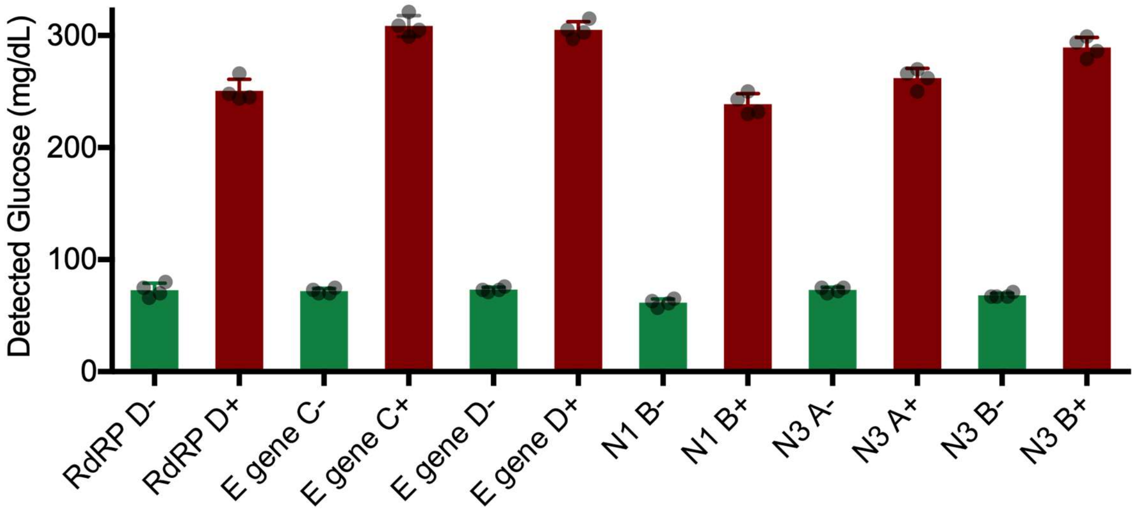

Supp. Fig. 7. Glucose meter output of SARS-CoV-2 toehold switches linked to a lactase reporter gene, using lactose as a substrate. CFS reactions were incubated for 2 hours. Toehold switch N3 B is SARS-CoV-2 sensor used in main text. The specificity of toehold switches is indicated on x-axis for SARS-CoV-2 RdRP, E gene and N gene as described in Fig. S6. N=4 technical replicates (as indicated by dot plots), data are presented as mean values +/- SD.

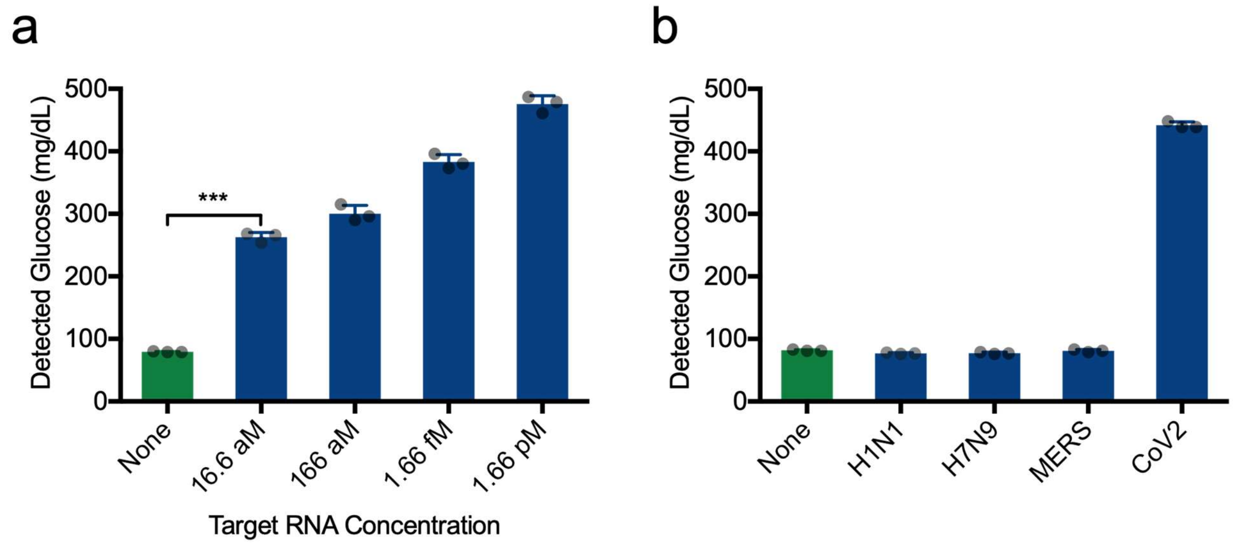

Supp. Fig. 8. Glucose meter output of SARS-CoV-2 toehold switch targeting the E gene (E gene C from previous figure). **a** Sensor sensitivity demonstrated with purified viral RNA. The concentration indicated reflects the viral RNA concentration of the 1-μL aliquot added to NASBA isothermal amplification reactions prior to glucose generation. ***: p=0.0005. **b** The SARS-CoV-2 gene E sensor specificity was tested using viral RNA genomes isolated from H1N1, H7N9, MERS and SARS-CoV-2 using an initial RNA concentration of 1.66 pM. N=3 technical replicates (as indicated by dot plots), representative of 3 independent experiments. Data are presented as mean values +/- SD.

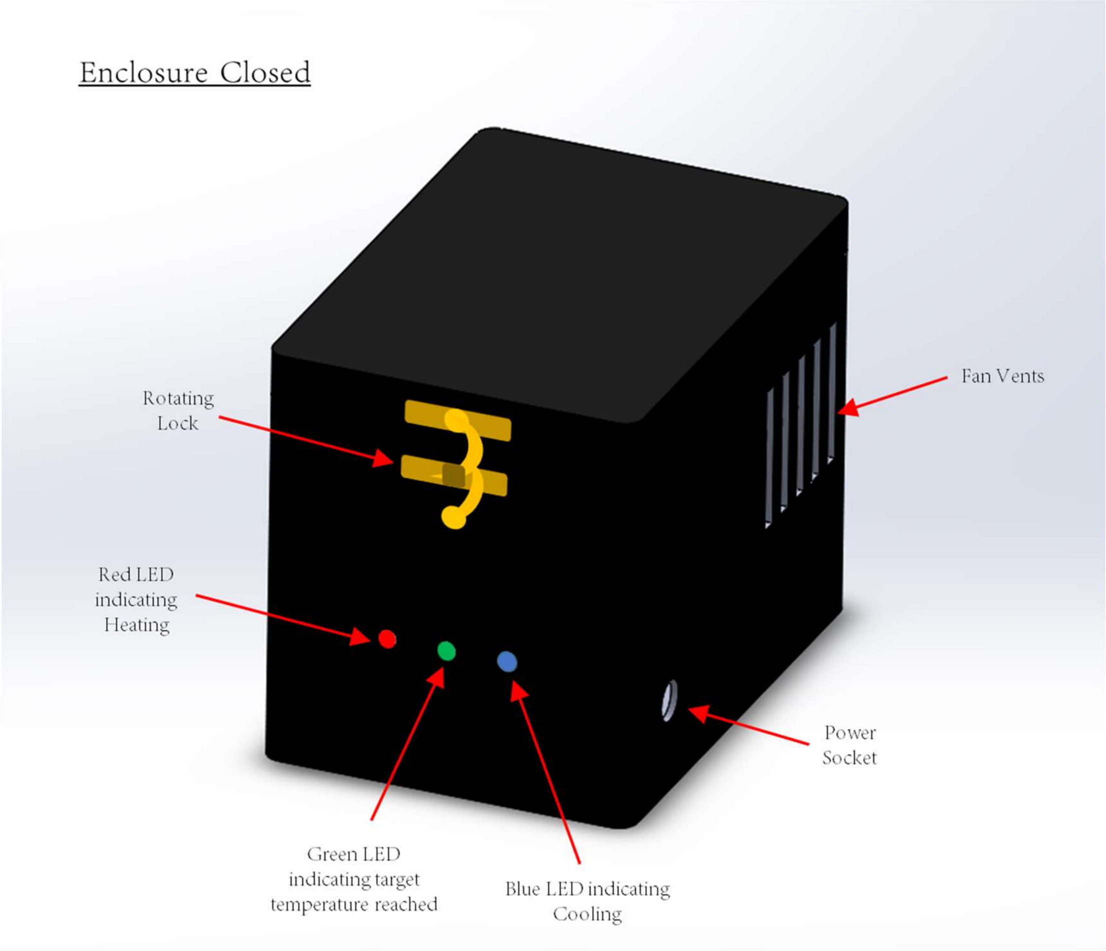

Supp. Fig. 9. A schematic of the companion incubator enclosure. The enclosure has two main components – the base and the lid. The material used to print the enclosure is polycarbonate, which has a heat deflection temperature and a glass transition temperature around 140°C. Off-the-shelf hook-latch and hinge are used to connect and close the lid and the base. Red, green and blue LEDs on the front of the box are illuminated to reflect heating, correct temperature and cooling status of the device, respectively.

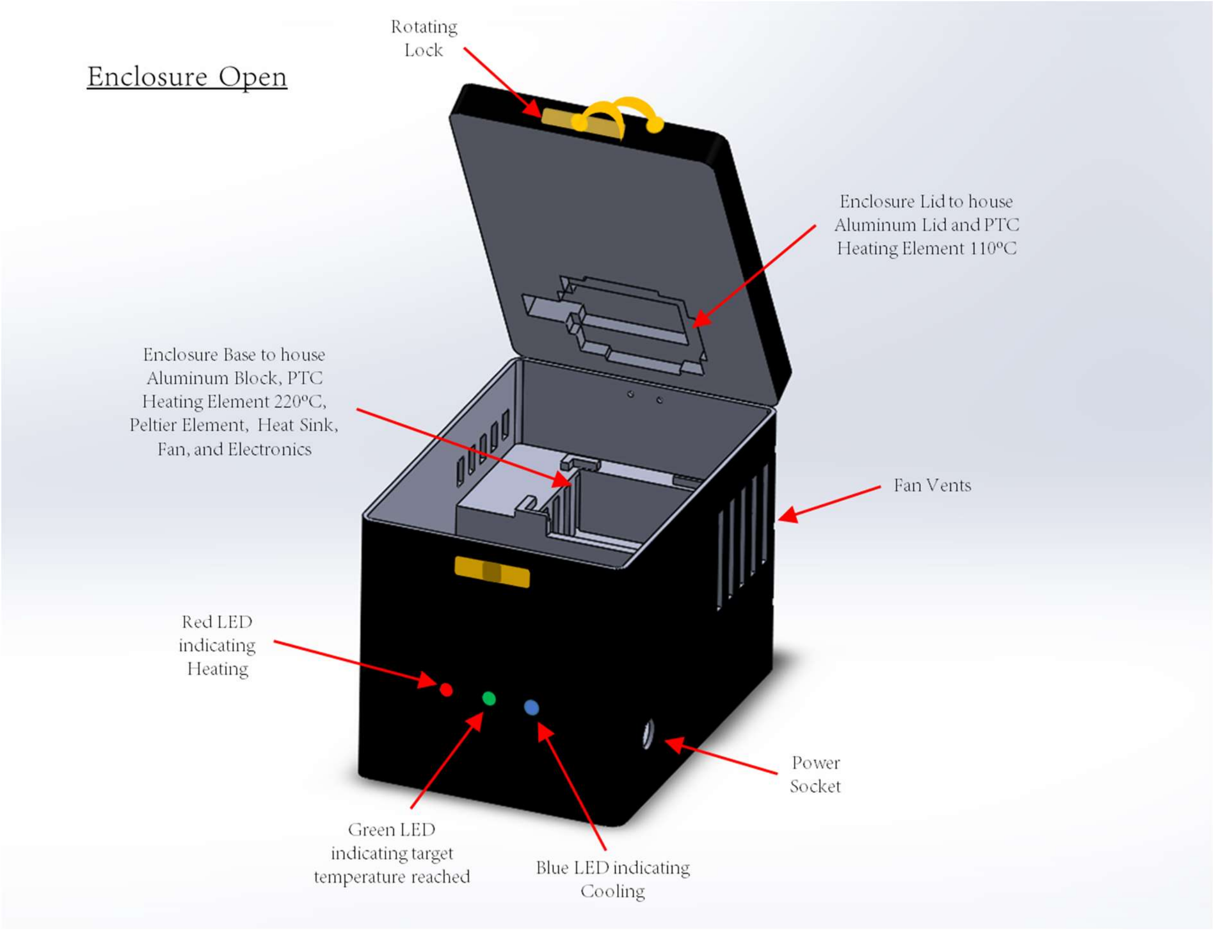

Supp. Fig. 10. A schematic of the companion incubator interior. Upon closing the enclosure lid, the heated aluminum lid is put in contact with the reaction tubes inside the aluminum block to prevent condensation during incubation. The enclosure base houses the components of the incubator, including the heated aluminum block for incubating the tubes and the electronic controls (see the circuit diagram, Supplementary Fig. 12).

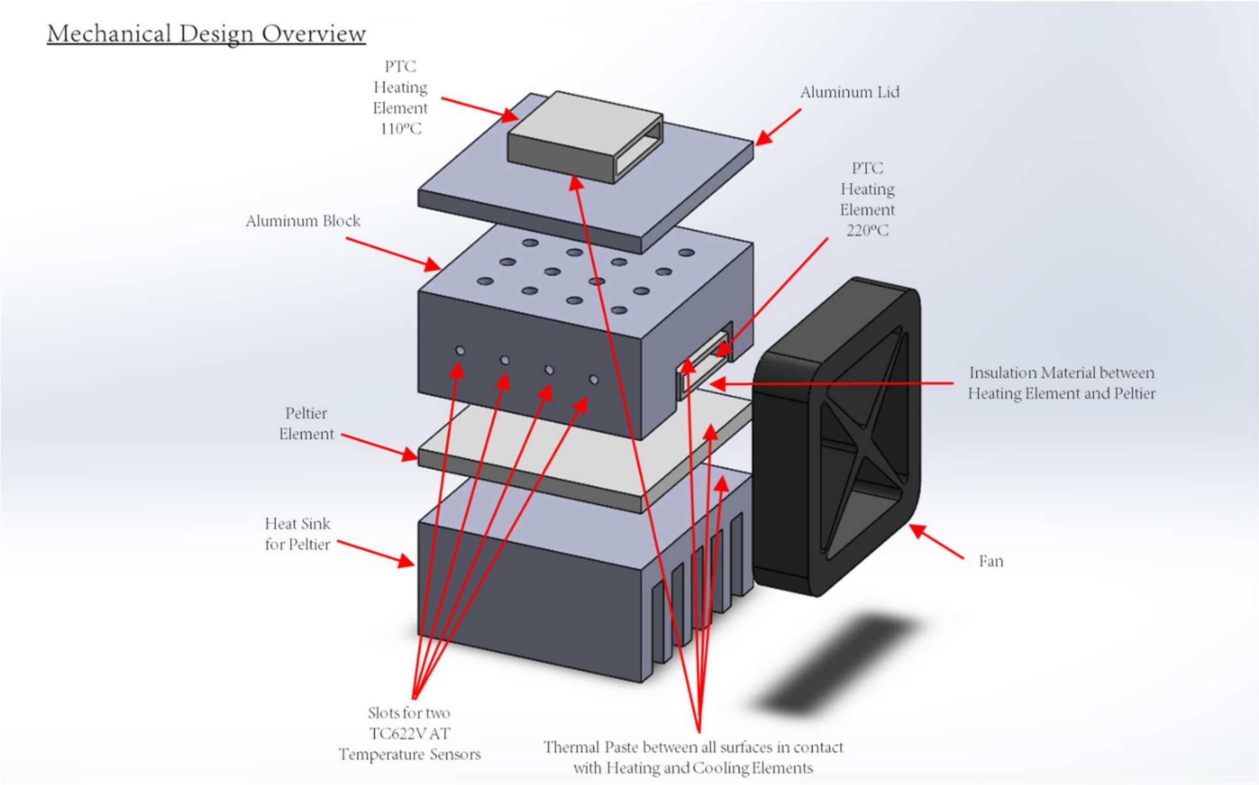

Supp. Fig. 11. A schematic of the heating and cooling components of the incubator. Two heating elements are involved – one for the lid, which is set to 110°C to prevent condensation in tubes, and one for the aluminum block to incubate the tubes at the four different temperature settings (37, 41, 65, and 100 degrees Celsius). A fan is used for the cooling steps. A Peltier element is used for thermoelectric cooling, and a heat sink is in contact with it. Insulating material is placed between the 220°C heating element and the Peltier element. Thermal paste is used at all points where a heating or cooling element contacts another component. Temperature sensors for detecting heating and cooling are attached to the aluminum block.

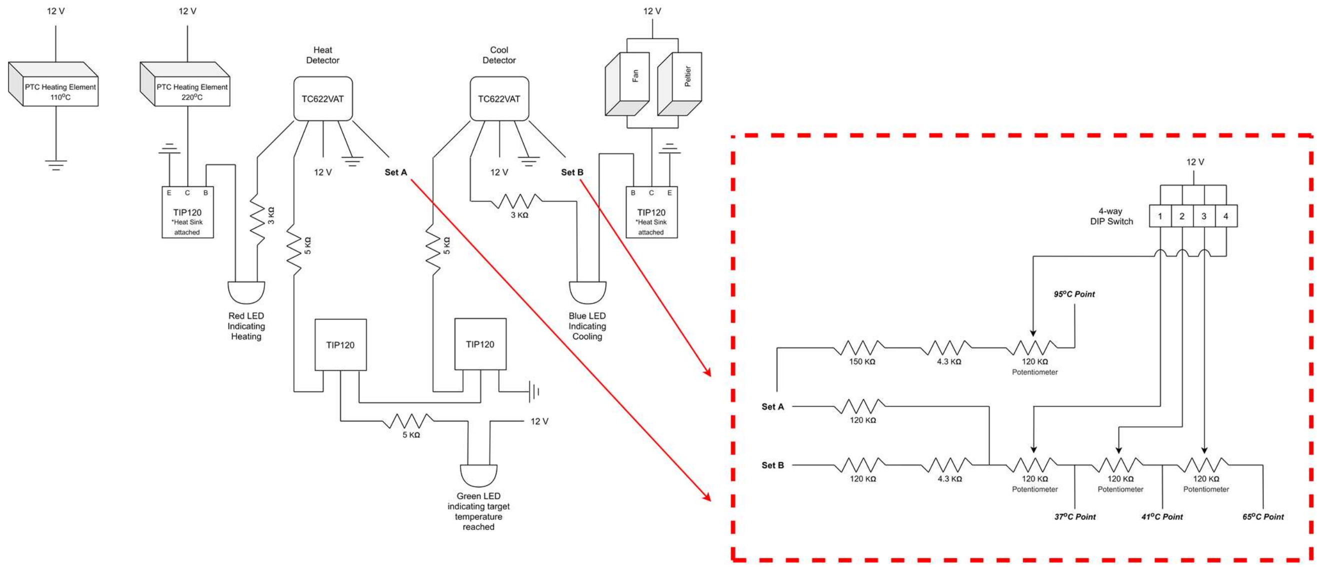

Supp. Fig. 12. The circuit diagram of the companion incubator. A 4-way DIP switch is used to determine the temperature of the incubator.

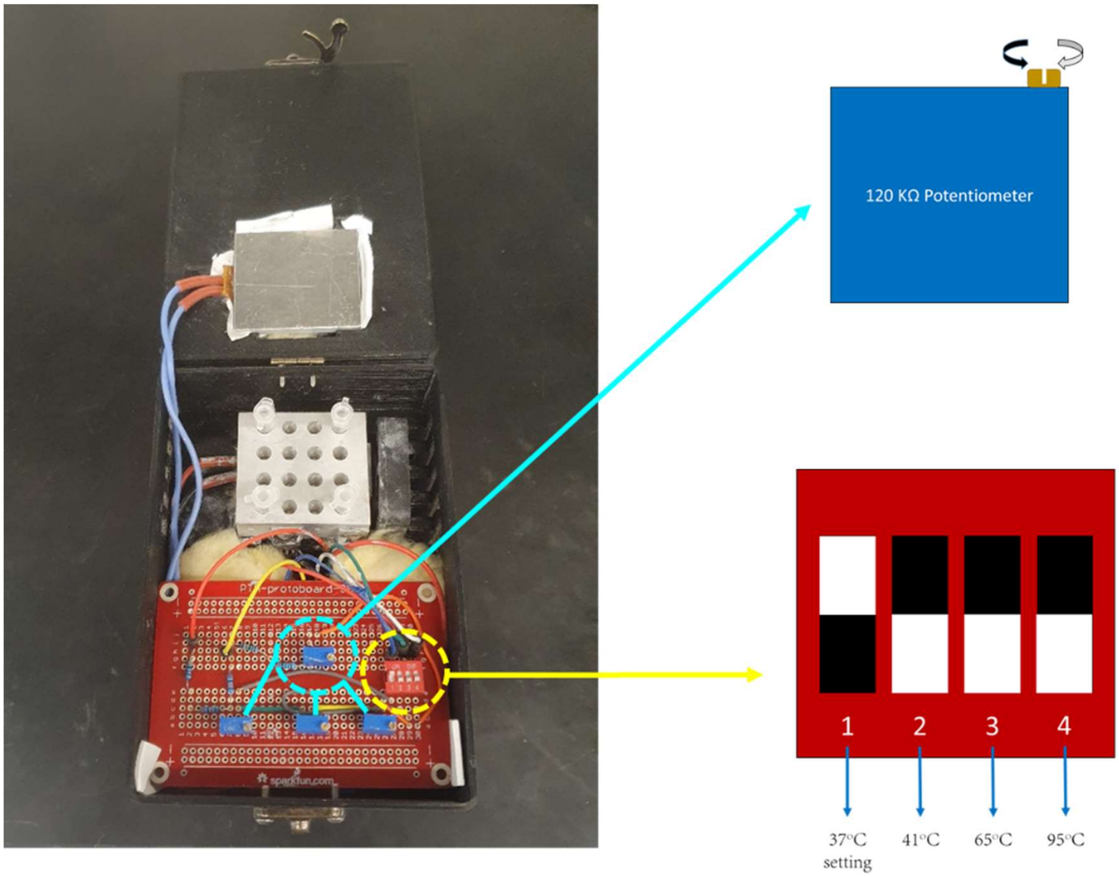

Supp. Fig. 13. Temperature control and selection system of the companion incubator. Each position on the switch corresponds to a temperature setting. Users can transition between incubation temperatures (37°, 41°, 65° and 95° C), by selecting the corresponding switch (red panel). Each switch is calibrated to the appropriate temperature during the device assembly process through adjustment of the associated potentiometer (knob rotated clockwise to decrease resistance and temperature, rotated counter-clockwise to increase resistance and temperature).
