# Supplementary material for: A glucose meter interface for point-of-care gene circuit-based diagnostics
Source: Nat Commun. 2021 Feb 1;12:724. doi: 10.1038/s41467-020-20639-6 (PMC7851131; doi:10.1038/s41467-020-20639-6)
Supplement: Supplementary file 4 — Description of Additional Supplementary Files [file 41467_2020_20639_MOESM4_ESM.pdf]

**Title:** Supplementary Data 1.
**Description:** List of the enzymes used and their corresponding genes. Table includes information on the source organism, gene sequence, and accession number.

**Title:** Supplementary Data 2.
**Description:** List of the DNA target sequences for the toehold switches used in experiments. When transcribed into RNA, these sequences act as triggers for their respective toehold switch.

**Title:** Supplementary Data 3.
**Description:** List of the novel toehold switches used in this study. The name of the toehold switch is provided along with the gene it targets and its sequence.

**Title:** Supplementary Data 4.
**Description:** List of the PCR primers used to amplify DNA encoding toehold switches. The amplicon sequence is given along with the sequences of the forward and reverse primers.

**Title:** Supplementary Data 5.
**Description:** List of NASBA primers used to amplify target RNAs. Sequences are given for forward and reverse primers
